# Probabilistic Modeling and Prediction of Continuous FRP Degradation Curves Based on CSDI Diffusion Models

**DOI:** 10.3390/polym18050587

**Published:** 2026-02-27

**Authors:** Yuan Yue, Ming-Li Zhou, Hui Shen, Wen-Wei Wang, Lei Zhang, Jing-Xian Shi, Bai-Chun Liang

**Affiliations:** 1 School of Civil Engineering and Architecture, Henan University, Kaifeng 475004, China; yueyuan@seu.edu.cn; 2 Department of Bridge Engineering, School of Transportation, Southeast University, Nanjing 211189, China; 3 Xuzhou Traffic Engineering General Contracting Co., Ltd., Xuzhou 221000, China; 4 ITS Research Center, School of Transportation, Southeast University, Nanjing 211189, China; 5 Faculty of Civil Engineering and Mechanics, Kunming University of Science and Technology, Kunming 650500, China; sara_shivip@163.com

**Keywords:** FRP, durability, continuous degradation curves, diffusion model, probabilistic modeling, conditional imputation

## Abstract

Traditional FRP durability forecasting predominantly treats performance evolution as a discrete “point-to-point” regression, inherently overlooking temporal coherence and stochastic uncertainty. This study proposes a novel probabilistic framework based on the CSDI diffusion model to reconstruct continuous FRP degradation curves. By formulating long-term forecasting as a conditional imputation task, the methodology generates physically consistent performance trajectories from sparse experimental observations. Results from a multi-factor database demonstrate that CSDI enables a paradigm shift to continuous sequence generation, achieving high predictive accuracy (RMSE = 0.332, R^2^ = 0.86) and robust probabilistic calibration (CRPS = 0.170) at a 30% missing ratio. This approach establishes a reliable probabilistic risk envelope, providing a scientific tool for the life-cycle reliability assessment of FRP structures under small-sample constraints.

## 1. Introduction

Fiber-reinforced polymer (FRP) bars have been extensively utilized in civil engineering reinforcement, marine structures, and aerospace sectors, owing to their high specific strength, light weight, and superior corrosion resistance [1,2]. However, under harsh service environments—such as strong alkalinity, salt spray, and hygrothermal cycles—FRP inevitably undergoes physicochemical degradation [3]. Accurately predicting the performance evolution and obtaining reliable degradation curves are of paramount importance for ensuring the life-cycle safety of infrastructure and developing scientific maintenance strategies [4,5,6,7]. Although accelerated aging tests have been widely implemented, achieving robust long-term performance predictions based on limited and discrete experimental data under the coupled effects of complex environmental factors remains a core challenge in current durability research [8,9,10,11,12].

Current durability prediction methods primarily rely on empirical models or data-driven machine learning (ML) approaches. Empirical models, typically represented by the Arrhenius equation, are physically interpretable and conform to degradation kinetics under idealized conditions [13,14,15,16], but they often struggle to capture complex nonlinear behaviors arising from coupled environmental and material factors. In contrast, ML-based methods, including Artificial Neural Networks (ANN) and ensemble models such as Random Forest or XGBoost [17,18,19], exhibit strong fitting capability and have been increasingly applied in cementitious materials research. Recent ANN-based studies have explored hybrid PINN–CatBoost predictors for rubberized steel-fiber concrete, ANN combined with RSM/ANOVA for hair-reinforced concrete, and CNN-assisted numerical modeling for aerated concrete porosity and thermal conductivity [20,21,22]. Despite their success, most existing ML approaches formulate durability prediction as a deterministic point-to-point regression problem under relatively regular measurements [23,24,25,26], overlooking the intrinsic temporal coherence of degradation evolution. This limitation becomes particularly pronounced when dealing with irregular sampling and substantial missing observations, where point-based predictions may fail to provide physically continuous trajectories or uncertainty-aware assessments.

To address these challenges, this study introduces a probabilistic forecasting framework based on the Conditional Score-based Diffusion Model (CSDI) [27,28]. Fundamentally different from traditional ML methods [29,30,31,32], the CSDI model adopts the complete performance degradation curve as the fundamental modeling unit rather than isolated data points. By learning the score function of degradation trajectories during the diffusion-denoising process, the model achieves a paradigm shift from “discrete point regression” to “continuous sequence generation.” This approach ensures that the generated prediction curves are logically continuous and smooth, enabling the spontaneous, physically plausible degradation trends consistent with FRP degradation laws. More importantly, CSDI inherently possesses uncertainty-handling capability, reconstructing the probabilistic space of performance evolution from complex stochastic samples, thereby maintaining strong generalization capabilities even under small-sample constraints.

In this study, an extensive experimental dataset of FRP durability was compiled through a literature review to evaluate the proposed CSDI framework. By transforming the long-term performance prediction into a conditional imputation task for irregularly sampled time series, the model leverages the diffusion-denoising process to generate continuous degradation curves from discrete observations. The primary focus of this study is to examine the model’s ability to enable physically plausible degradation trends and quantify stochastic uncertainties under small-sample constraints. This work aims to bridge the gap between discrete experimental data and continuous performance evolution, providing a more scientific and transparent decision-support tool for the reliability assessment of FRP structures.

## 2. Problem Definition

**Figure 1:**
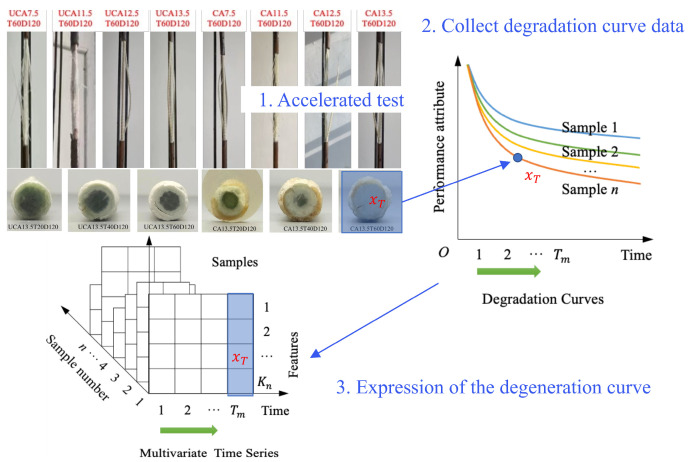
Definition of the continuous degradation curve prediction problem.

This study models the performance degradation process of FRP as a conditional stochastic process prediction problem. In FRP durability tests, the measured mechanical performance indicators (such as residual strength and elastic modulus) can be regarded as a stochastic sequence evolving with exposure time t. Here, the exposure time refers to the physical testing time at which measurements are taken and should be clearly distinguished from the algorithmic time index used later in the diffusion-based learning process. As illustrated in Figure 1, under given environmental factors K (e.g., temperature, solution concentration, and stress level), the degradation behavior of each specimen is governed by an underlying continuous degradation curve, while experimental measurements provide only finite and discrete observations sampled from this curve. Accordingly, the experimentally obtained observation sequence can be expressed as $X^{ob}=\{x_{0},\;x_{1},\;\ldots \;x_{t},\;{\ldots \;x}_{T}\}$, where ${\;x}_{T}$ denotes the material performance state at exposure time T.

Unlike traditional deterministic point prediction methods (which only learn a pointwise mapping from time t to performance y), this study treats the performance degradation of FRP as a continuous degradation curve and uses the entire degradation trajectory as the fundamental modeling unit. The prediction task is defined as learning the conditional probability distribution $P(Y|X^{ob},K)$, where:

- Y represents the model-generated continuous degradation path spanning the entire service life;
- $X^{ob}$ denotes the experimentally obtained, discontinuous, and finite set of observed samples;
- K represents environmental constraint variables that regulate the degradation rate and evolutionary trend.

During inference, the model generates a set of representative performance evolution trajectories through multiple random samplings, thereby constructing a probabilistic space of performance degradation. To comprehensively evaluate the accuracy and reliability of the generated curves from a probabilistic perspective, this study introduces the Continuous Ranked Probability Score (CRPS) as the core evaluation metric. By quantifying the discrepancy between the predicted cumulative distribution function (CDF) and the true experimental observations [27], CRPS effectively assesses the model’s ability to capture degradation patterns under conditions of limited sample size and measurement noise.

## 3. Methodology

### 3.1. Modules Design

The modules include dataset construction, data preprocessing, and control constraint training modules. Among them, the data preprocessing module is responsible for formatting the experimental samples collected in the literature review and finally generating 3D graphical datasets. This step must be done before the first task begins, as it requires learning the distribution of pixels (i.e., “attribute points”) across the entire three-dimensional sequence structure. The second-generation task is performed jointly by the second and third modules under the guidance of the interpolation training strategy. The interpolation strategy introduces specific conditions into the model as “control constraints,” so that the loss function is concentrated on the attribute points with prediction demand, thus improving the model’s predictive ability for samples under certain conditions. Therefore, before applying the interpolation strategy, it is also necessary to divide the data structure into two parts: “specific conditions” and “predicted requirements”. However, there is a problem with this strategy: the actual predicted requirements may be missing. Therefore, a simulation dataset is constructed using the simulation strategy, and the “ground-truth” is provided with the “simulation predicted requirements” as a training target. Three modules are then run to complete the training process and two generation tasks, and the strategy is verified to be feasible—if the model’s prediction results for the simulated structure are acceptable, then the model can be considered reliable for the real data structure.

**Figure 2:**
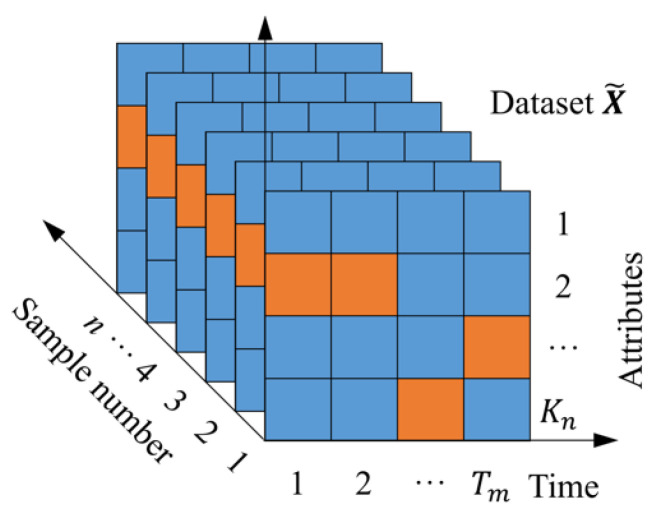
Three-dimensional matrix sequence.

The modules’ design fully combines the advantages of a three-dimensional matrix structure. Three-dimensional sample matrix structure refers to the representation of samples in the form of a two-dimensional matrix, and the samples are transformed into images with attribute points as pixels. All samples in the dataset are arranged in the same direction to form a three-dimensional matrix sequence, where the third dimension represents the sample number, similar to a picture gallery (as shown in Figure 2). Using a 3D matrix series to represent the dataset makes performance prediction more attractive—it provides more comprehensive and detailed degradation information; at the same time, it is also easy to seamlessly integrate with deep learning technology and use its image-like structural characteristics for further mining and analysis.

### 3.2. Dataset Construction Module

#### 3.2.1. Sample Collection

As mentioned earlier, many scholars have conducted research on the durability performance of FRP bars in corrosive environments. However, the focus of each research work is different, and the experimental results are not comprehensive. Therefore, it is necessary to establish a dataset that includes the experimental results of all scholars, making this dataset widely representative. To establish this dataset, the material parameters, environmental factors that affect the residual performance of FRP bars, as well as the residual strength, elastic modulus, and micro damage of FRP bars after a period of durability, are considered as independent attributes, and each attribute is assigned a symbol *K_i_* (*i* = 1, *… n*), as shown in Table 1. Here, *n* represents the number of FRP bar attributes.

Similarly, durability time can also be considered as an independent parameter to form a time series in chronological order. If the durability time length is assumed to be *T*, a symbol $T_{j}$ (j= 1, …m) can also be assigned, in which *m* represents the durability time point. Therefore, by using *K* as the column vector and *T* as the row vector, we can obtain a sample matrix *X* with dimensions $K_{i}\times T_{j}$, which reflects the dataset formed by a certain attribute of FRP bar changing with durability time. For example, an element *x_ij_* in the matrix *X* represents the *i*-*th* attribute value of FRP reinforcement at the *j*-*th* durability time point.

It should be noted that the sample matrix X is a single sample formed for one scholar’s experimental project. For experimental projects involving multiple scholars or multiple materials of FRP bar, a dataset can be used to represent them. If the number of experimental samples is N, it can form the dataset $\tilde{X}$.

#### 3.2.2. Three-Dimensional Matrix Dataset and Uniform Formatting

**Figure 3:**
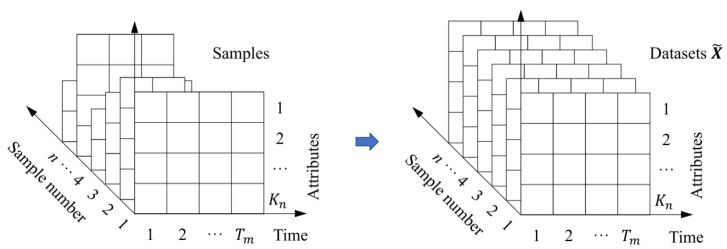
Schematic of normalized datasets $\tilde{X}$.

The collected 3D matrix sequences need to be uniformly formatted (see Figure 3) to ensure that all samples have the same measurement meaning in the same location. In this way, location-based feature learning has a clear measurement meaning. The result is a sequence of three-dimensional matrices, each of which records a specific condition and time information. The evolution of condition and performance is recorded in the sample dimension.

**Figure 4:**
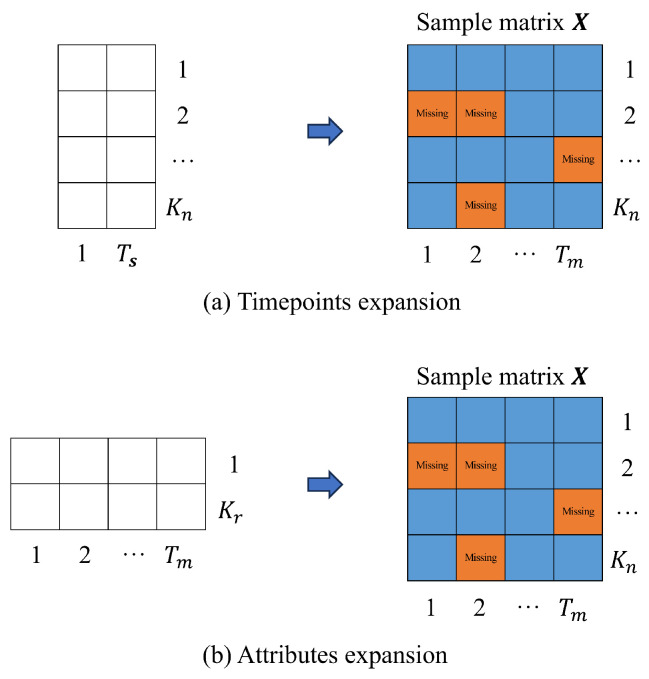
Normalized sample matrix format.

The normalized format should ensure that all samples conform to a uniform standard. The “standard” here refers to the number of points in time recorded in the dataset as the length of the time dimension and the number of attributes defined as the length of the attribute dimension. In practical applications, the experimental sample size obtained by scholars is often smaller than the standard size (i.e., R≤n, s≤m). Therefore, it is necessary to extend the sample bidirectionally in time and attribute directions when performing the normalization. Specifically, the time vector is added in the time direction, the attribute vector is added in the attribute direction, and the missing position generated by the extension of the mask tag is missed (as shown in Figure 4).

### 3.3. Data Preprocessing Module

#### 3.3.1. Dataset Dividing Based on Simulation Strategy

**Figure 5:**
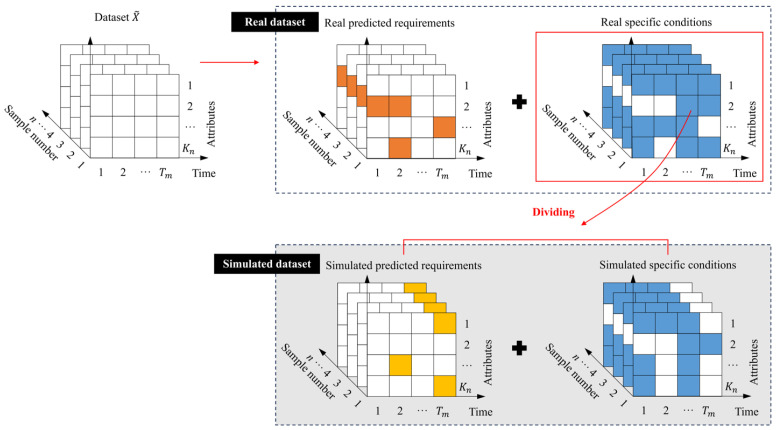
Divide the specific conditions and predicted requirements.

The first function of the data preprocessing module is to simulate the structural features of the original dataset, divided into “specific conditions” and “predicted requirements” by adjusting the “missing ratio”. By adjusting the missing ratio, the ratio of the two parts of the simulation dataset can be changed flexibly. This provides the ground-truth for the training target. In the “predicted requirements” of the original dataset, there are two types of missing values with different physical meanings: first of all, in the independent sample with known test results, all specific conditions cannot be completely covered, resulting in the inevitable loss of residual strength data of FRP bars under certain conditions or at a certain point in time; in addition, in the example where only specific conditions are known but not tested, so the performance attribute value is completely missing. Figure 5 shows the dividing results.

#### 3.3.2. Mark Dividing Results with Mask

The second function of the data preprocessing module marks the segmentation results. To accommodate different types of training targets, two distinct labeling strategies are employed in this study. The first strategy directly identifies the positions of the predicted requirements in the real dataset, while the second strategy determines the number of simulated predicted requirements proportionally based on a given missing ratio φ and randomly selects corresponding known positions in the dataset. The mask matrix *M* maintains the same shape as the sample matrix *X*, with its elements taking values of 0 or 1. Specifically, assuming $M\;=\;\{m_{1:K,1:L}\}\;$ belongs to $\;{\left\{0,\;1\right\}}^{K\times L}$, $m_{k,l\;}=0$ if $x_{ij}$ represents a predicted requirement and $m_{k,l\;}=1$ if $x_{ij}$ represent a specific condition.

The missing ratio is defined as the proportion of missing attribute values relative to the total number of attribute values in the dataset, as given in Equation (1).

(1) $$\varphi =\frac{N_{{\tilde{x}}^{ta}}}{N_{{\tilde{X}}^{ob}}}\times 100\%$$

where $N_{{\tilde{x}}^{ta}}$ is the number of attribute values in the simulated predicted requirement ${\tilde{x}}^{ta}$, and $N_{{\tilde{X}}^{ob}}$ is the number of attribute values in the simulated dataset ${\tilde{X}}^{ob}$.

**Figure 6:**
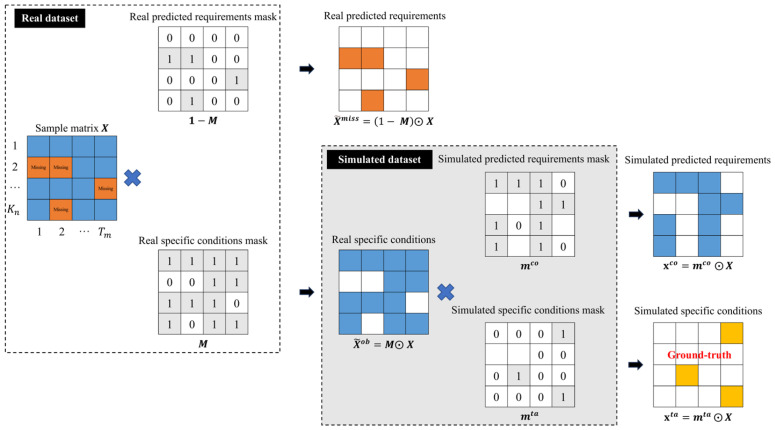
Mark dividing results with mask.

According to the above labeling method, two types of predicted requirements and their corresponding positions can be obtained by dot multiplying the mask matrix with the real dataset *X*. For example, the calculation $\left(1-\tilde{M}\right)⊙\tilde{X}$ can obtain the real predicted requirements and their position (${\tilde{X}}^{miss}$). Similarly, by calculating the dot product between the simulation-predicted requirements mask and the dataset $\tilde{X}$, the simulation-predicted requirements (${\tilde{x}}^{ta}={\tilde{m}}^{ta}⊙\tilde{X}$) can be obtained. Similarly, two specific conditions can be obtained in a similar way. The symbols for all mask tags and all dividing results are shown in Figure 6 and Table 2.

#### 3.3.3. Clarification on Padding and Smoothing

It is emphasized that the above masking operation is also used to handle padded entries introduced when organizing sequences of different lengths into a unified tensor. Padded values serve only as placeholders and are always paired with the corresponding mask entries; they do not contribute to the loss or gradient updates. Therefore, the padding process does not impose any artificial smoothing or functional-form assumption on degradation rates. In sparsely observed regions, uncertainty is reflected by a wider predictive distribution rather than deterministic interpolation.

### 3.4. Control Constraint Training Modules for Interpolation

#### 3.4.1. Training Module with Auxiliary Variables

Next is describing the design details of the training module to implement the first-generation task, which is to generate new samples that conform to the degradation pattern of the original dataset. This module adopts the architecture of the diffusion model [27,33,34,35] and introduces serialization auxiliary variables to transform the complex high-dimensional learning task into a relatively simple step-by-step learning task.

The auxiliary variables are constructed by gradually adding Gaussian noise of different intensities to the original dataset $\tilde{X}$, resulting in a sequence of noisy data representations. Specifically, the forward diffusion process is defined as:

(2) $${\tilde{X}}_{s}={\tilde{X}}_{s-1}+\epsilon_{s},\;\;\epsilon_{s}∼N\left(0,\sigma_{s}^{2}I\right),\;t\in \{1,\cdots ,S\}$$

where $\epsilon_{t}$ denotes Gaussian noise with zero mean and variance $\sigma_{t}^{2}$, and *I* is the identity matrix. Under this framework, the distribution of the auxiliary variables $q\left({\tilde{X}}_{1:S}\right)$ and the corresponding noise distributions are analytically tractable. By exploiting these known distributions and their inherent symmetries, the model learns to reverse the diffusion process, mapping auxiliary variables back to the original data space and thereby implicitly learning the data distribution $p(\tilde{X})$. This reverse learning mechanism not only simplifies the training procedure but also improves the quality and stability of generated samples.

Here, the diffusion step index s (with s = 1, …, S) denotes an algorithmic time variable used for probabilistic denoising in the diffusion model, rather than the physical exposure time of material degradation. Accordingly, the noise schedule $\sigma_{s}$ is a purely algorithmic construct and does not correspond to any physical degradation rate or kinetic parameter.

**Figure 7:**
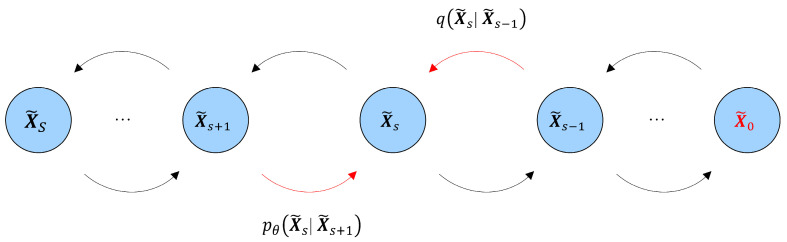
Series structure of auxiliary variables and original dataset. Note that the diffusion steps illustrated here correspond to the auxiliary denoising process in the model, not to the actual physical aging or exposure timeline.

**Figure 8:**
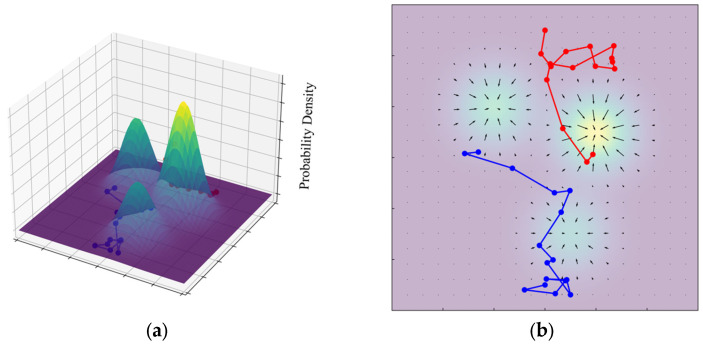
Langevin dynamics-based sampling trajectories: (**a**) three-dimensional Gaussian mixture distribution with sampling trajectories; and (**b**) probability density field with corresponding sampling trajectories.

The core idea of the reverse learning process is to minimize the *Kullback–Leibler* (KL) divergence between the learned reverse transition distribution and the known forward diffusion process, as expressed in Equation (3). By progressively denoising the noisy variable ${\tilde{X}}_{s}$ toward ${\tilde{X}}_{s-1}$, the model learns the underlying data distribution. To enable efficient sampling from this high-dimensional and complex distribution, Langevin dynamics is employed, and the corresponding update rule is given in Equation (4). The structure of the auxiliary variable sequence used during training and the sampling trajectory generated by Langevin dynamics are illustrated in Figure 7 and Figure 8, respectively. The original clean dataset is denoted as ${\tilde{X}}_{0}$.

(3) $$KL\left(p_{\theta }\left({\tilde{X}}_{s-1}|{\tilde{X}}_{s}\right)∥q\left({\tilde{X}}_{s}|{\tilde{X}}_{s-1}\right)\right)$$

(4) $${\tilde{X}}_{s-1}={\tilde{X}}_{s}-\eta \nabla_{{\tilde{X}}_{s}}logp_{\theta }\left({\tilde{X}}_{s}\right)+\sqrt{2\eta }\zeta$$

where: ${\tilde{X}}_{s}$ t is the data of the current time step; η is the learning rate; $\nabla_{{\tilde{X}}_{s}}logp_{\theta }\left({\tilde{X}}_{s}\right)$ is the gradient data log probability (score); and ζ∼N(0,I) is a standard normal distribution of noise.

The score function $\nabla_{{\tilde{X}}_{s}}logp_{\theta }\left({\tilde{X}}_{s}\right)$ is learned by training a neural network $s_{\theta }\left({\tilde{X}}_{s},s\right)$. To avoid explicitly computing gradients in high-dimensional space, which may lead to numerical instability, the learning objective is reformulated as a noise prediction problem. The resulting loss function is defined as Equation (5):

(5) $$L\left(\theta \right)=E_{s,{\tilde{X}}_{0}}\left[{∥\nabla_{{\tilde{X}}_{s}}\log p_{\theta }\left({\tilde{X}}_{s}\right)-s_{\theta }\left({\tilde{X}}_{s},s\right)∥}^{2}\right]\;=\;E_{s,{\tilde{X}}_{0},\epsilon }\left[{∥\epsilon -\epsilon_{\theta }\left({\tilde{X}}_{s},s\right)∥}^{2}\right]$$

where ${\tilde{X}}_{0}$ denotes the original dataset; ${\tilde{X}}_{s}$ is the noisy auxiliary variable at time step *s*; ϵ is Gaussian noise injected during the forward diffusion process; and $\epsilon_{\theta }\left({\tilde{X}}_{s},s\right)$ is the noise predicted by the neural network.

#### 3.4.2. Control Constraint of Specific Condition Attributes

The second task of this study focuses on inferring unknown performance values in new samples under specific conditions, which can be formulated as a conditional time-series interpolation problem. To incorporate conditional information, the loss function is extended as shown in Equation (6), where optimization is restricted to the artificially masked positions corresponding to the predicted requirements. This design enables the model to specifically learn how to reconstruct missing values rather than fitting the entire sequence. The conditional loss remains based on mean squared error (MSE) between the predicted noise and the actual injected noise, as further detailed in Equation (7).

(6) $$L\left(\theta \right)=E_{s,{\tilde{X}}_{0}}\left[{∥\nabla_{{\tilde{X}}_{s}}logp_{\theta }\left({\tilde{X}}_{s}|{\tilde{x}}^{ta}\right)-s_{\theta }\left({\tilde{X}}_{s},s,{\tilde{x}}^{ta}\right)∥}^{2}\right]$$

(7) $$L\left(\theta \right)=E_{s,{\tilde{X}}_{0},\epsilon }\left[{∥\epsilon -\epsilon_{\theta }\left({\tilde{X}}_{s},s\right)∥}^{2}⊙{\tilde{m}}^{ta}\right]$$

where ${\tilde{x}}^{ta}$ denotes the artificially masked target values used for conditional training, and ${\tilde{m}}^{ta}$ is the corresponding binary mask indicating missing positions.

#### 3.4.3. Algorithmic Detail

Algorithm 1 summarizes the complete sample-level procedure of the proposed CSDI-based framework, including training optimization (Steps 1–6) and conditional inference (Steps 7–12), where observed entries are preserved via masking, and only missing values are imputed.
**Algorithm 1:** CSDI-Based Durability Imputation Framework.$\mathrm{Input}:\;\mathrm{Observed}\;\mathrm{data}\;\tilde{X}$, Mask m, Conditions c, Total diffusion steps *S*Part 1: Training (Optimization)1: Repeat until converged:2:$\mathrm{Sample}\;\mathrm{data}\;\mathrm{batch}\;X_{0}∼\tilde{X}$ and time step s∼Uniform (1,…,S)3:Sample noise ϵ∼N(0,I)4:$\mathrm{Corrupt}\;\mathrm{data}:\;X_{s}=\sqrt{\bar{\alpha_{s}}}X_{0}+\sqrt{1-\bar{\alpha_{s}}}\epsilon$5:$\mathrm{Predict}\;\mathrm{noise}:\;\epsilon_{\theta }\left(X_{s},s|c\right)$6:Update parameters θ$\;\mathrm{by}\;\mathrm{minimizing}\;\nabla_{\theta }∥\epsilon -\epsilon_{\theta }∥$Part 2: Inference (Conditional Imputation)7: $\mathrm{Initialize}\;X_{S}∼N(0,I)$ (Pure Gaussian Noise)8:For s=S,…,1 do:9:$\mathrm{Predict}\;\mathrm{noise}\;\epsilon_{\theta }$$\;\mathrm{and}\;\mathrm{estimate}\;X_{s-1}\;$(Denoising step)10:Conditioning (Imputation): $X_{s-1}^{co}=\sqrt{{\bar{\alpha }}_{s-1}}X^{ob}+\sqrt{1-{\bar{\alpha }}_{s-1}}\epsilon^{\prime }$(Re—noise observed data)11:$\mathrm{Combine}:\;X_{s-1}=X_{s-1}⊙(1-m)+X_{s-1}^{\mathrm{co}\;}⊙m$12:End For$\mathrm{Output}:\;\mathrm{Imputed}\;\mathrm{sample}\;X_{0}$

Notation Clarification: To ensure mathematical precision, we explicitly distinguish between dataset-level and sample-level representations.

❿ Uppercase tilde symbols (e.g., $\tilde{X}$) denote the complete dataset (a collection of N standardized samples).
❿ Uppercase non-tilde symbols (e.g., X) denote an individual standardized degradation trajectory represented in matrix form and processed within the algorithm (Algorithm 1 operates on sample-level matrices, not raw experimental records).
❿ Dimensions: the input matrix X in Algorithm 1 has dimensions K×T, where K denotes the number of attributes and T denotes the sequence length (number of exposure-time points).
❿ Algorithm 1 operates on standardized sample matrices constructed as described in Section 3.2.2, rather than on raw experimental records (x).

## 4. Experiments

This chapter is divided into two parts, application design and result presentation, aiming to demonstrate how to achieve the research objectives of this paper. The application design uses graphical modeling to combine the characteristics of durability data to improve the sample structure to achieve two advantages: one is to contain multiple performance attributes in the same sample, which can predict the tensile strength and modulus at the same time; a second advantage is the integration of damage-related conditional information to guide training by incorporating damage information into the sample as a performance attribute as well. The results presentation provides case study results, including not only performance evaluation of the model but also sample results generated under specific conditions (i.e., research objectives). In order to further verify the advanced nature of the model, the prediction results of the degradation mechanism are also analyzed.

### 4.1. Dataset Construction

The key to simultaneously predicting multiple performance attributes and guiding damage information lies in the personalized 3D matrix sequence. In this case, the attributes are expanded to encompass comprehensive attributes, such as tensile strength, tensile modulus, and damage information within each sample. These comprehensive performance attributes and their corresponding specific conditions were derived from a dataset compiled from 43 published studies. The complete list of source publications used for dataset construction is summarized in Table A1. The dataset covers a wide range of specific conditions, including aging temperatures ranging from 20 °C to 80 °C; aging durations ranging from 0 to 720 days; different environmental conditions involving acid, salt water, and alkali solutions at various pH levels; as well as sustained load levels ranging from 0% to 50%. To ensure the reliability and integrity of the dataset during the implementation process, strict sample validation and cleaning procedures were conducted in order to eliminate outliers and duplicate entries.

Specifically, the tensile tests under each set of specific conditions are presented uniformly according to the matrix form in Table 3. Each sample matrix consists of 14 attributes (*K* column vectors) and 54 test exposure-time points (*T* row vectors), arranged in *K × T*. The 14 attributes include 11 specific conditions and 3 performance attributes. Among them, the three performance attributes are tensile strength, tensile modulus, and micro-damage. The 11 specific conditions are divided into two categories: corrosion environment factors and material parameters: 6 items are related to corrosion environment, such as exposure time, temperature, stress level, pH value, solution salt concentration, and concrete water-cement ratio; there are five material parameters, including fiber type, resin type, fiber content, surface state, and diameter. It is important to note that the 54 time points are the total number of all exposure times taken from the dataset.

### 4.2. Training Module with Damage-Attention

#### 4.2.1. Damage Attribute

**Figure 9:**
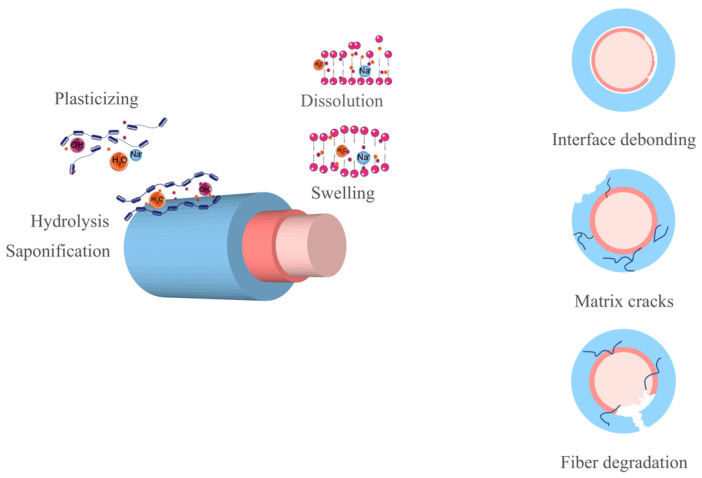
Three types of damage to FRP bars in corrosion solutions.

In the dataset construction module, corrosion detection techniques were reviewed [36,37,38,39,40]. Statistical corrosion detection results were based on three identifiable damage types corresponding to composite composition. The three types of damage are shown in Figure 9:

Damage Type 1: Interface debonding is caused by the actions of hydrolysis, saponification, and plasticization. These chemical processes can lead to the breakdown of the bond between the fiber and the matrix, resulting in interface debonding, which is crucial for load transfer.

Damage Type 2: matrix cracks are caused by swelling and dissolution; dissolution compromises the interface, and swelling can lead to increased internal pressures, and dissolution can weaken the matrix, both contributing to the development of matrix cracks.

Damage Type 3: fiber degradation directly affects the tensile strength of FRP bars, as the fiber is the main stressed component.

#### 4.2.2. Attention Guided with Damage Attribute

**Figure 10:**
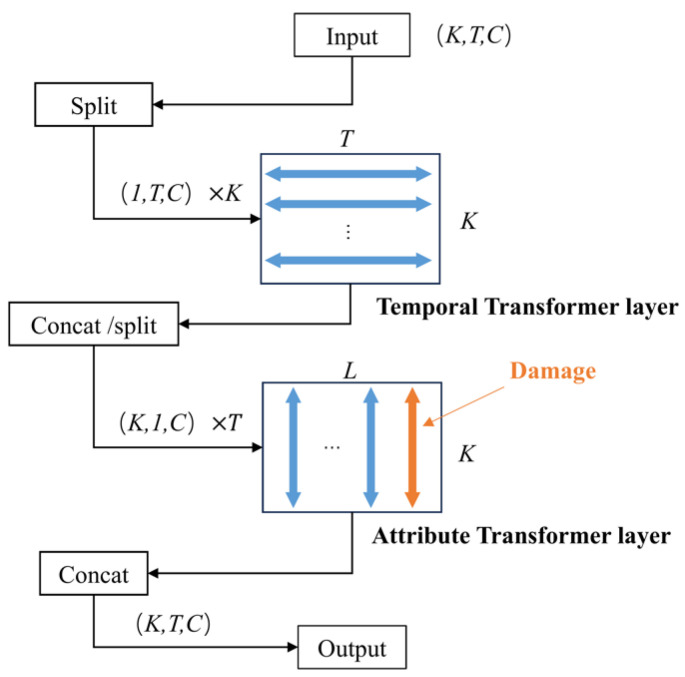
Improved attention structure.

By encoding damage information into damage column vectors and feeding them into a transformer-based multi-layer attention structure (Figure 10), the model is better able to learn complex dependencies between time and attributes. Specifically, in the preprocessing stage, four damage features (three damage types + no damage) and their corresponding exposure time are encoded as damage column vectors, which provide additional conditional context during model training through two attention modules: time transform layer and attribute transform layer. The time transform layer captures the time dependence of each feature, and the attribute transform layer deals with the dependence between different features, so that the model can better capture the statistical dependencies between time and attribute dimensions under heterogeneous and sparsely observed conditions.

## 5. Result and Discussion

### 5.1. Training Details and Hyperparameters

In order to solve the problem of inaccurate evaluation indicators of small data sets, this study adopted the K-fold cross-validation method [41]. Specifically, the dataset was randomly divided into five equal-sized subsets (each containing 282 sample points for a total of 1410 sample points and 407 sample matrices). One subset was selected as the verification set, and the other four subsets as the training set. This process is repeated five times, and the results of the five iterations are averaged to determine the most accurate training model. During the training, the batch size was set to 16, the number of training rounds was set to 200, the learning rate was set to 0.001, and the ReLU activation function was used. The accuracy of the validation set was evaluated every five training rounds.

To implement these training strategies and evaluation metrics, PyTorch 1.9.0 was used as an open-source deep learning framework, with the operating system being Linux, CUDA version 10.1, and Python version 3.7.0. Hardware configurations included an NVIDIA GeForce RTX 3090 GPU (NVIDIA, Santa Clara, CA, USA) and an Intel Xeon(R) Silver 4114 CPU (Intel Corporation, Santa Clara, CA, USA). By carefully designing the training strategies and evaluation indicators, this study fully exploited the potential of limited small datasets and made a meaningful exploration to improve the performance and robustness of the model.

### 5.2. Performance Evaluation of the Models

Nine experiments were conducted to evaluate the effects of different missing ratios on the model performance and to find the model with the best performance, setting the φ range between 10% and 90%. According to the evaluation results, the best-performing configuration under the current dataset and training setup is selected.

#### 5.2.1. Missing Ratio Test

The optimization results of the model based on the change in missing ratio show that when the missing ratio is between 20% and 30%, the prediction results of tensile strength are most consistent with the experimental results, and the model performance is the best. The evaluation results are shown in Table 4, including root mean square error (RMSE), mean absolute error (MAE), and regression coefficients of strength (R^2^). The data show that RMSE and MAE decrease first and then increase during the process of increasing the missing ratio. When the missing ratio is between 20% and 40%, the RMSE is less than 0.4; when the number of data points is between 10% and 40%, the MAE is less than 0.2. However, after the data point missing ratio exceeds 50%, RMSE and MAE rise sharply, and the R^2^ value drops below 0.7. This indicates that too high a missing ratio may cause the whole feature of the sample to dissipate, while too low a missing ratio may affect the training effect of the interpolation method.

#### 5.2.2. Performance Evaluation of Selected Models

**Figure 11:**
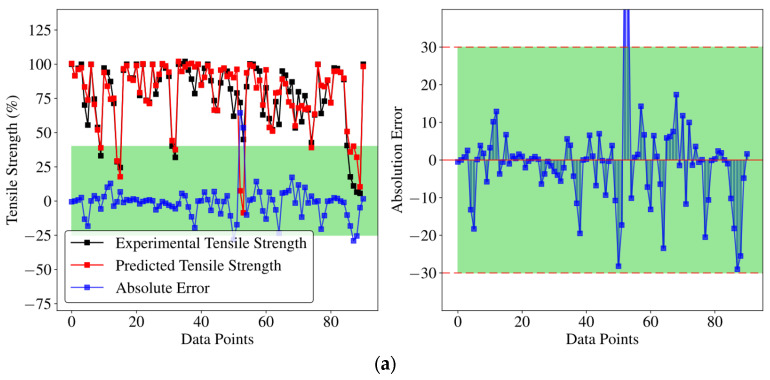

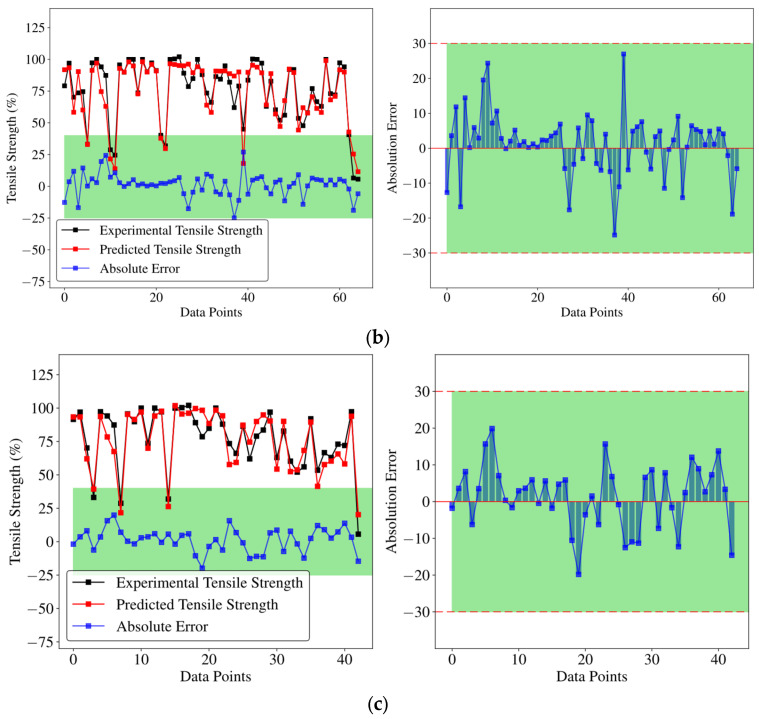
Prediction performance and error analysis under different missing ratios: (**a**) missing ratio = 40%; (**b**) missing ratio = 30%; and (**c**) missing ratio = 20%. Left: comparison between experimental and predicted tensile strengths; Right: corresponding absolute error distributions.

**Figure 12:**
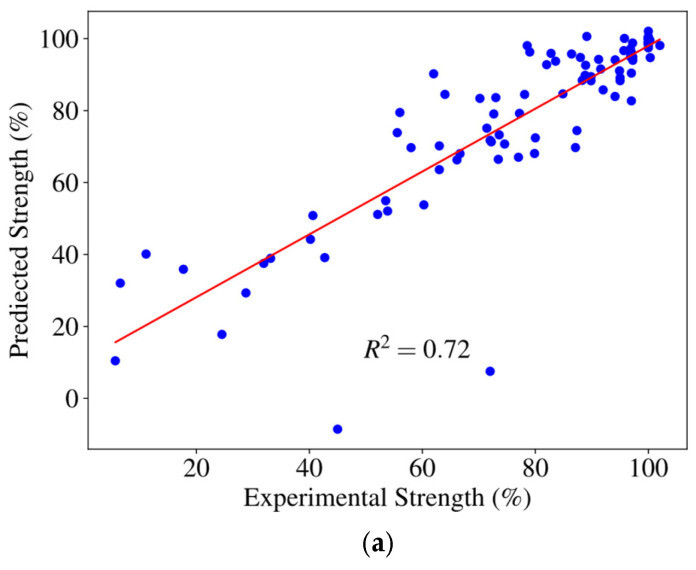

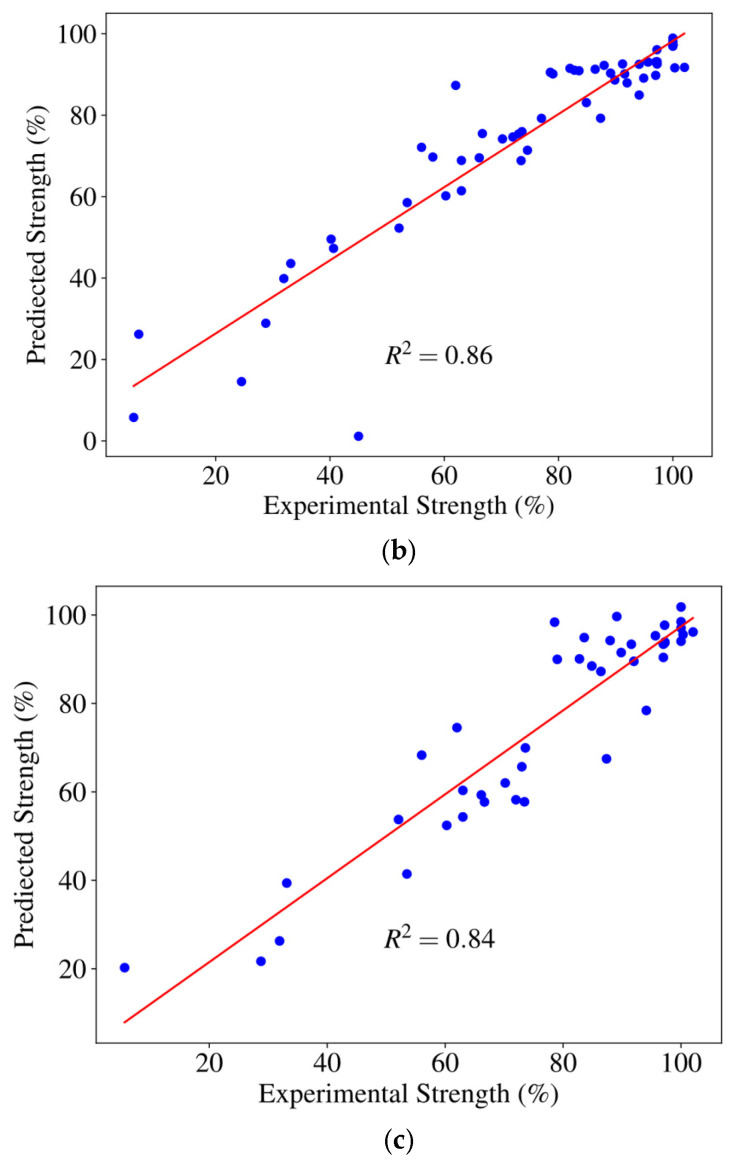
Prediction accuracy evaluation under different missing ratios: (**a**) missing ratio = 40%; (**b**) missing ratio = 30%; and (**c**) missing ratio = 20%. Scatter plots compare predicted and experimental tensile strengths, with the corresponding R^2^ values indicated.

In Figure 11 and Figure 12, the best-performing configurations under the current setup (φ = 20%, 30%, 40%) are shown. Figure 11 shows intensity comparison and error analysis, with experimental and predicted intensity comparison on the left and absolute error on the right. In the 40% missing ratio model (Figure 11a), the strength of the prediction was basically consistent with the experimental strength, and the absolute error did not fluctuate much, with 93% of the data points having an error of less than 20%. In contrast, the absolute error fluctuation of the other two models is smaller, and the prediction accuracy is higher. Furthermore, Figure 12 and Table 5 compare the model fitting and its performance with other studies [23,25,42], respectively. Overall, the model performed well, demonstrating its reliability and data analysis capabilities, maintaining good predictive performance in complex data environments and demonstrating flexibility and stability when dealing with different specific conditions.

### 5.3. Probabilistic Generation of Continuous Degradation Curves

To fulfill the objective of predicting continuous FRP degradation curves, the selected models were deployed to reconstruct performance evolution from discrete experimental data. The Continuous Ranked Probability Score (CRPS) was employed to evaluate the probabilistic modeling accuracy. By conducting 150 sampling iterations for each time step, the model approximates the stochastic range of material states.

As shown in Table 6, the CRPS remains below 0.25 when the missing ratio is between 10% and 40%, demonstrating high reliability in probabilistic modeling under data-constrained conditions.

**Figure 13:**
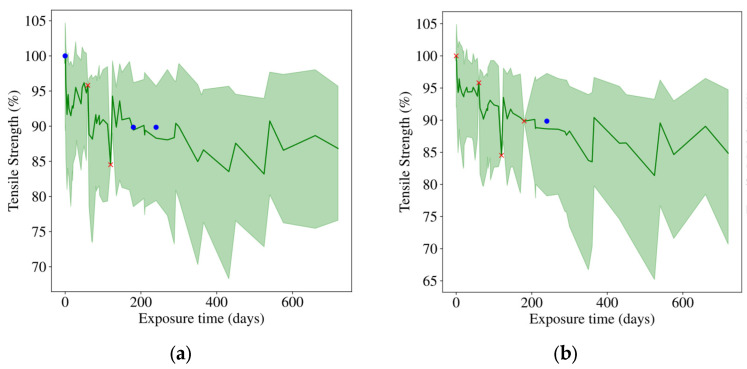
Sample generation under specific conditions: (**a**) missing ratio of 40%; (**b**) missing ratio of 30%.

Figure 13 visualizes the generated samples at missing ratios of 30% and 40%. The solid green line (mean prediction) tracks the tensile durability trend, while the generated sample paths bridge the temporal gaps between discrete observations. This suggests that the proposed model can transform sparse data into continuous, physically plausible degradation curves and achieve the probabilistic prediction goal defined in this study.

Notably, the model learns a conditional distribution of degradation trajectories rather than enforcing strict monotonicity; therefore, the mean/median curve may show mild local fluctuations under sparse and heterogeneous observations, whereas the overall tendency is reflected by the distribution envelope (a monotonic post-processing step can be applied if required).

## 6. Conclusions and Limitations

This study establishes a robust probabilistic modeling framework for the continuous FRP degradation curves by leveraging the CSDI diffusion models. By transforming long-term durability forecasting into a conditional imputation task, the proposed methodology successfully bridges the gap between sparse, discrete experimental observations and the continuous performance evolution profiles required for engineering safety assessments.

Experimental validation demonstrates that the CSDI-based architecture effectively captures the intrinsic temporal coherence and complex nonlinear characteristics under coupled environmental factors. At a representative missing ratio of 30%, the model achieves high-fidelity reconstruction with an RMSE of 0.332, R^2^ of 0.86, and a CRPS of 0.170. This paradigm shift—from traditional point-to-point regression to continuous sequence generation—enables the model to naturally follow physically plausible degradation trends without the constraints of predefined empirical functions. Furthermore, the model’s inherent ability to quantify stochastic uncertainty provides a transparent probabilistic risk envelope for material performance under small-sample constraints.

Despite these advantages, three limitations remain. First, the random masking used for self-supervised training may not fully represent systematic experimental gaps (e.g., lack of long-term measurements), although the model reflects this distinction via widened predictive intervals. Second, quantitative baselines against unified mechanistic kinetics (e.g., Arrhenius) were not feasible due to the high heterogeneity and non-isothermal nature of the literature-compiled dataset, which violates strict kinetic assumptions. Third, damage types were simplified using one-hot encoding, overlooking the complexity of coexisting mechanisms. Future work will prioritize controlled evaluations under structured missingness, the integration of explicit mechanistic monotonicity constraints, and the exploration of multi-label damage representations.

In summary, this research advances the application of generative deep learning in composite durability and provides a practical, data-driven framework for probabilistic prediction of long-term degradation sequences under sparse and heterogeneous observations.

## Figures and Tables

**Table 1:** Attributes and timepoints of the sample matrix.

	Name	Symbol	Number	Content
Attributes	Material parameters	$K_{i}$	i=1, …a	FRP diameter, etc.
	Environmental factors	$K_{i}$	i=a+1, …b	Alkaline, salt, etc.
	Residual performance	$K_{i}$	i=b+1, …n	Strength, etc.
Timepoint	Time point	$T_{j}$	j=1, …m	/

**Table 2:** Mark of real and simulated datasets.

Name	Symbol	Mask
Real dataset	$\tilde{X}$	1
Real predicted requirement	${\tilde{X}}^{miss}$	$1-\tilde{M}$
Real specific condition	${\tilde{X}}^{ob}$	$\tilde{M}$
Simulated dataset	${\tilde{X}}^{ob}$	$\tilde{M}$
Simulated predicted requirement	${\tilde{x}}^{ta}$	${\tilde{m}}^{ta}$
Simulated specific condition	${\tilde{x}}^{co}$	${\tilde{m}}^{co}$

**Table 3:** Sample matrix form with *K* attributes and *T* timepoints (*K* = 14, *T* = 54).

Specific Conditions											Performance Attributes		
Corrosive Environments Factor						Material Parameters							
Exposure Time	Temp	Stress	pH	Salt-Ion	W/C	Fiber Type	Resin	Fiber Content	Surface	Diameter	Strength	Modulus	Damage
T1													
T2													
…													
T52													
T53													
T54													
A1	A2	A3	A4	A5	A6	A7	A8	A9	A10	A11	A12	A13	A14

Ai denotes the i-th attribute, and Tj  denotes the j-th time point.

**Table 4:** Evaluation results with different missing ratios in the testing set.

Missing-Ratio φ	10%	20%	30%	40%	50%	60%	70%	80%	90%
RMSE	0.523	0.363	0.332	0.417	0.597	0.602	0.655	0.766	0.818
MAE	0.194	0.163	0.141	0.180	0.304	0.288	0.313	0.391	0.455
$R^{2}$ (strength)	0.87	0.84	0.86	0.72	0.62	0.52	0.66	0.25	0.20

**Table 5:** Comparison of predictive performance with other studies.

Ref.	Model	R^2^
Iqbal et al. [42]	GEP	0.84
Kaloop et al. [25]	Minimum Probabilistic Machine Regression (MPMR); ANFIS-GA; DNN;	0.93
Khan et al. [23]	ANN model with Gray Wolf optimization method	0.88
30% missing-ratio	Diffusion model with condition (this study)	0.86
20% missing-ratio	Diffusion model with condition (this study)	0.84

**Table 6:** Evaluation results with different missing ratios in the testing set.

Missing-Ratio φ	10%	20%	30%	40%	50%	60%	70%	80%	90%
CRPS	0.240	0.200	0.170	0.212	0.341	0.327	0.352	0.432	0.517

## Data Availability

The data presented in this study are available on request from the corresponding author due to practical considerations related to data organization and documentation.

## Abbreviations

The following abbreviations are used in this manuscript:

CSDI	Conditional Score-based Diffusion Model
FRP	Fiber-reinforced Polymer
ML	Machine Learning
CRPS	Continuous Ranked Probability Score
CDF	Cumulative Distribution Function
ANN	Artificial Neural Networks
XG-Boost	Random Forest

## Appendix A

### Appendix A.1. Source Publications Used for Dataset Construction

These comprehensive performance attributes and their corresponding specific conditions were derived from a dataset compiled from 43 published studies. The dataset was constructed based on experimental data extracted from the following publications:

**Table A1:** Source publications used to compile the dataset.

Ref. No.	Author	Year	FRP Type	Exposure/Environment
[2]	Sawpan	2016	GFRP	Alkaline solution, temperature
[3]	Al-Salloum	2013	GFRP	Harsh environments
[7]	Ali	2018	GFRP	Resin-dependent environments
[10]	Ali	2019	CFRP	Sustained load, severe environment
[12]	Ali	2019	BFRP	Alkaline environment
[13]	Ali	2020	BFRP	Long-term exposure
[15]	Andreea	2015	BFRP	Aging conditions
[43]	Arczewska	2021	GFRP	Concrete environment
[44]	Benmokrane	2020	BFRP/GFRP/CFRP	Alkaline solution
[45]	Benmokrane	2016	CFCC	High temperature, alkaline
[46]	Benmokrane	2017	GFRP	Concrete environment
[47]	Benmokrane	2015	GFRP/BFRP	Alkaline solution
[48]	Chen	2006	GFRP	Accelerated aging
[49]	Chen	2007	GFRP	Accelerated aging
[50]	Chen	2017	GFRP	Soil, alkaline exposure
[51]	Chuan	2011	GFRP	Alkali, acid, salt
[52]	Chunhua	2016	FRP	Water, seawater, alkaline
[53]	Davalos	2012	GFRP	Concrete environment
[54]	El-Hassan	2018	GFRP	Seawater concrete, sustained load
[55]	Elgabbas	2015	BFRP	Concrete environment
[56]	Fergani	2018	GFRP	Severe environments
[57]	Gang	2015	BFRP	Sustained load, corrosive solution
[58]	Gang	2015	BFRP	Alkaline environment
[59]	Jin	2020	GFRP	Load + alkaline solution
[60]	Katsuki	1995	FRP	Alkali attack
[61]	Khatibmasjedi	2020	GFRP	Seawater concrete
[62]	Kim	2008	GFRP	Environmental exposure
[63]	Liu	2019	BFRP	Wet–dry alkali–salt cycles
[64]	Lu	2021	BFRP	Seawater sea-sand concrete
[65]	Manalo	2020	GFRP	Concrete & simulated environments
[66]	Micelli	2004	FRP	Concrete environment
[67]	Morales	2021	GFRP	Seawater concrete
[68]	Rifai	2020	BFRP	Alkaline & moist concrete
[69]	Robert	2010	GFRP	Extreme temperature
[70]	Robert	2010	GFRP	Preloading
[71]	Robert	2013	GFRP	Saline solution & concrete
[72]	Robert	2010	FRP	Temperature acceleration
[73]	Roh	2017	GFRP	Concrete alkalinity
[74]	Ruiz Emparanza	2022	GFRP	Marine environments
[75]	Tu	2020	GFRP	Sustained load & environment
[76]	Wang	2017	BFRP/GFRP	Seawater & sea-sand concrete
[77]	Wang	2018	BFRP/GFRP	Sustained load & seawater
[78]	Won	2008	GFRP	Alkaline solution & water

## Appendix B

### Appendix B.1. Ablation Study on the Damage-Attention Module

To assess the contribution of the damage-attention module, we conduct an ablation study in which this module is removed while all other components of the network and training configurations are kept unchanged. The objective of this experiment is to evaluate whether incorporating damage-related information provides measurable benefits to probabilistic degradation modeling, rather than optimizing overall performance. Both the full model and the ablated variant are trained and evaluated under the same data split, training schedule, and evaluation protocol to ensure a fair comparison. As reported in Table A2, removing the damage-attention module leads to a moderate increase in RMSE and CRPS, indicating that damage information contributes positively to model accuracy while the overall framework remains stable.

The ablated model is trained and evaluated under the same data split, training schedule, and evaluation protocol as the full model.

**Table A2:** Both models are evaluated under the same training and evaluation protocol.

Model	RMSE	CRPS
Full model	0.332	0.170
w/o damage-attention	0.348	0.183
